# Stereotactic Radiosurgery: Fundamentals and A Historical Vignette

**DOI:** 10.7759/cureus.105856

**Published:** 2026-03-25

**Authors:** Maria Angelica Guevara, Juan Diego Zerpa Urdaneta, Valentina Zorro, Edgar G Ordóñez-Rubiano, Oscar Fernando Zorro Guio

**Affiliations:** 1 Neurosurgery, Fundación Universitaria de Ciencias de la Salud, Bogotá, COL; 2 Neurosurgery, Fundación Universitaria Juan N. Corpas, Bogotá, COL; 3 Neurosurgery, Pontificia Universidad Javeriana, Bogotá, COL; 4 Neurosurgery, Fundación Santa Fe de Bogotá, Bogotá, COL

**Keywords:** cyberknife, gamma knife, history of neurosurgery, linear accelerator, stereotactic radiosurgery

## Abstract

Stereotactic radiosurgery (SRS) is a minimally invasive neurosurgical technique that allows, through the administration of high doses of ionizing radiation, the treatment of intracranial lesions. A detailed review was carried out on its historical development, from the early origins of radiological technology to the physical foundations of ionizing radiation. The evolution of the main prototypes is described, such as the Gamma Knife, adapted linear accelerator (LINAC), and the robotic CyberKnife (Accuray Inc., Madison, WI, USA), emphasizing their technical characteristics, clinical results, and indications in clinical practice. Nowadays, SRS is a valuable complement to conventional neurosurgery, offering a safe and effective alternative for the treatment of lesions in eloquent brain areas, where open surgery would involve greater risks.

## Introduction and background

Discovery of X-rays and the foundations of ionizing radiation

Wilhelm Conrad Röntgen (1845-1923) conducted experimental research in cathode-ray physics that culminated in the discovery of X-rays on November 8, 1895. Shortly thereafter, he produced the first radiograph using his wife’s hand, marking the beginning of medical imaging [1].

In 1896, X-rays were first applied therapeutically for the treatment of cutaneous lesions and superficial neoplasms. In the same year, Henri Becquerel demonstrated spontaneous radiation emission from uranium salts, establishing the phenomenon of natural radioactivity. These findings were later expanded by Marie and Pierre Curie, who identified new radioactive elements such as polonium and radium in 1898 and formally introduced the concept of radioactivity as a property of atomic transformation [2].

Ionizing radiation interacts with biological tissues through atomic and molecular ionization, producing DNA damage that may lead to apoptosis or loss of cellular replicative capacity [3]. Among the different types of ionizing radiation, X-rays and gamma rays are high-energy photon radiation widely used in medical applications [4].

The subsequent introduction of cobalt-60 sources and the development of linear accelerator (LINAC) technology during the mid-twentieth century enabled the treatment of deep-seated tumors, establishing the technological and conceptual foundations for stereotactic radiosurgery (SRS) [5].

Methods

We performed a targeted literature search using PubMed, focusing on the history and physical fundamentals of stereotactic radiosurgery (SRS). Our scope spanned from the discovery of X-rays in 1895 to early 2026, prioritizing landmark developments in prototypes like the Gamma Knife, LINAC, and CyberKnife (Accuray Inc., Madison, WI, USA). We selected articles and clinical guidelines based on their relevance to core clinical indications, including brain metastases and vascular malformations. Finally, we synthesized these findings qualitatively to map the technological and clinical evolution.

## Review

Stereotactic radiosurgery (SRS)

Since the discovery of X-ray tubes and the subsequent use of cobalt-based radiation sources, radiation technology has progressively evolved and been optimized for therapeutic applications [6]. In 1908, Victor Horsley and Robert Clarke developed the first stereotactic frame using Cartesian coordinates for the localization of specific targets in the animal brain; however, its application in humans was not achieved until 1947 by Ernst A. Spiegel and Henry T. Wycis, largely due to the limitations in visualizing deep intracranial structures. This technique was applied in patients with severe psychiatric disorders, in whom the head was immobilized using a plaster cast; contrast material was subsequently injected into the cerebral ventricles, and radiographic images were obtained to localize deep brain nuclei that were believed at the time to be responsible for these conditions [6]. In parallel, the first particle accelerator was developed by R. Van de Graaff in 1922, although its clinical implementation was limited and short-lived around 1940 due to high costs. In 1943, the invention of the betatron introduced a high-energy X-ray-generating accelerator characterized by greater tissue penetration and reduced radiation scatter [6].

The Swedish neurosurgeon Lars Leksell, building upon prior technological advances, envisioned directing radiation beams to the brain with the precision of stereotactic techniques [6]. In 1951, together with Börje Larsson, he introduced the concept of radiosurgery to describe the destruction of a precisely defined intracerebral target using a single, focused radiation dose without open surgery. That same year, the first radiosurgical procedures were performed using convergent X-ray beams, initially applied in patients with functional disorders such as trigeminal neuralgia. Although these early treatments demonstrated focal tissue ablation without significant injury to adjacent structures, the limited penetration of X-rays restricted their application to relatively superficial lesions [6].

In 1935, John Lawrence and Cornelius A. Tobias in California performed proton irradiation of both healthy and pathological tissues, a technique they later termed the “atomic knife” in 1948, reflecting its capacity for selective destruction of intracerebral normal or pathological tissue. This approach enabled the generation of convergent proton beams using a cyclotron, analogous to the system proposed by Leksell for X-ray-based radiosurgery. In 1955, this technique was applied to perform hypophysectomy as a means of achieving hormonal blockade for palliative treatment in patients with breast cancer and osseous metastases [7].

In 1967, Leksell completed the construction of the first Gamma Knife prototype (gamma unit) at Sophiahemmet Hospital in Stockholm, Sweden. This system emitted millimetric gamma-ray beams that converged on a central focal point where the target lesion was positioned. The first clinical procedure was performed in October of that year in a patient with a craniopharyngioma. With subsequent advances in neuroimaging techniques, radiosurgery expanded to the treatment of vascular and neoplastic lesions, whereas its initial applications had been limited to thalamic targets, pain syndromes, and movement disorders [7].

In 1975, the Gamma Knife was installed at Karolinska Hospital in Stockholm. Two years later, in 1977, neurosurgeons Juan A. Barcia and José Solorio established the clinical use of radiosurgery for the management of arteriovenous malformation (AVMs), developing a novel technique based on cross-fired beams from cobalt-60 sources delivered through 35 fixed fields, specifically arranged to produce convergent irradiation. This approach enabled the selective delivery of high radiation doses to AVMs while preserving the surrounding healthy brain tissue [7].

In 1982 in Buenos Aires and later in 1986 in Paris, Victor E. Derechinsky and Osvaldo O. Betti, respectively, implemented and adapted the cross-fired beam technique using a LINAC. In parallel, third-generation Gamma Knife systems were installed, including one in Argentina by Bunge in 1984, another in England by Forester in 1985, and finally a unit in Pittsburgh in 1988 [7].

Subsequently, Elekta, founded by Leksell in 1972, developed the Gamma Knife Model B in 1996 and the Model C in 1999, incorporating automated collimator systems. Seven years later, in 2006, the platform was redesigned with the introduction of the Gamma Knife Perfexion (Elekta AB, Stockholm, Sweden), which expanded target coverage and treatment flexibility. In 2015, the integration of cone-beam computed tomography (CBCT) imaging with motion monitoring enabled dose fractionation, leading to the development of the Gamma Knife ICON model (Elekta AB, Stockholm, Sweden) [8,9].

**Figure 1:**
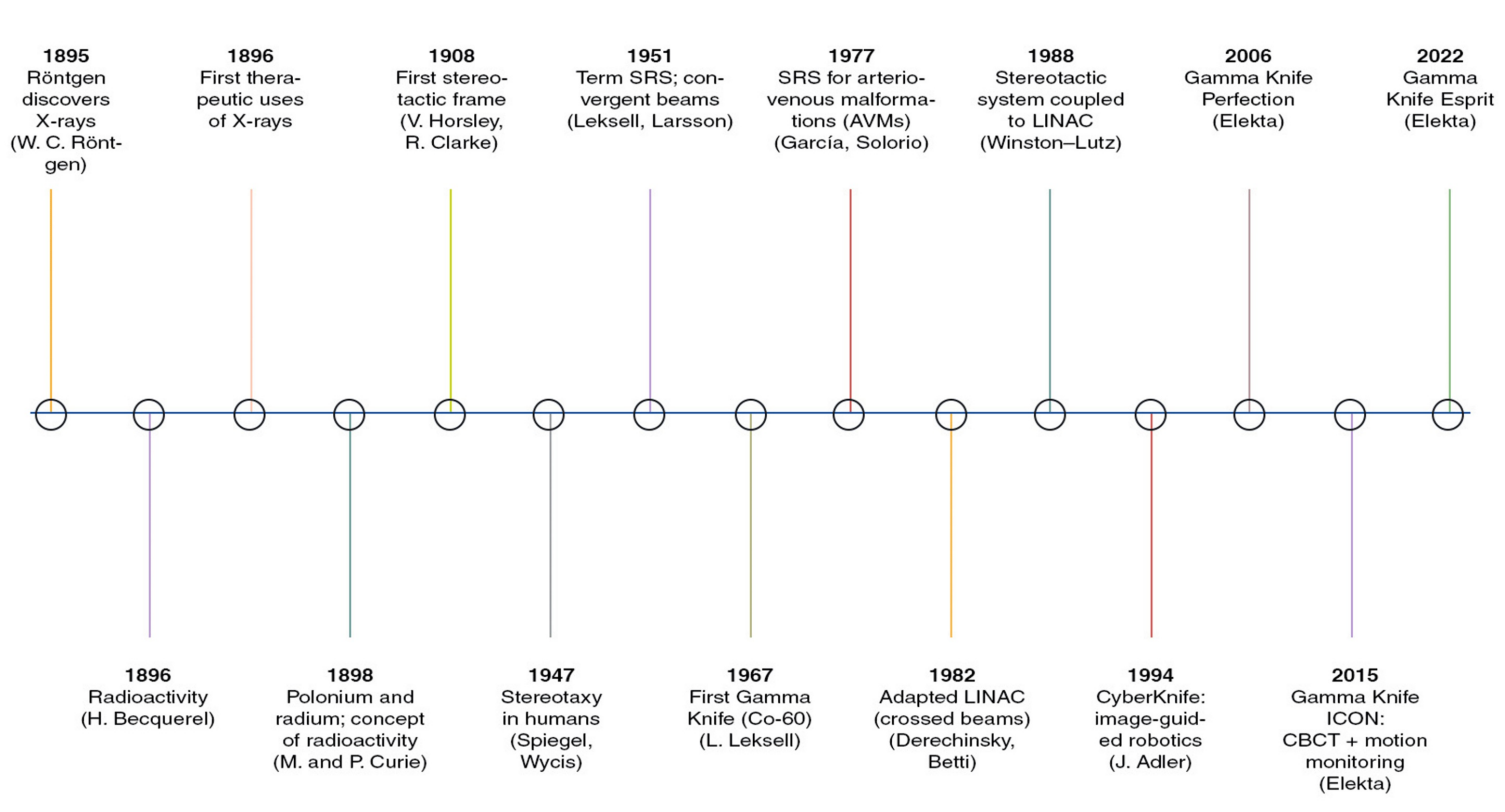
Timeline of key historical milestones in stereotactic radiosurgery (SRS). Figure created by the authors using Adobe Photoshop 2023 (Adobe Inc., San Jose, CA, USA). CBCT, cone-beam computed tomography; LINAC, linear accelerator.

In parallel, in 1969, proton beams generated by cyclotrons at Harvard were adapted for the treatment of AVMs, giving rise to stereotactic proton therapy (SPT). In 1988, Kenneth R. Winston and Wolfgang Lutz published the technical description of a stereotactic localization system coupled to a LINAC, and during the 1990s, Jay Steven Loeffler further advanced this approach with the development of fractionated stereotactic radiotherapy (FSRT) [10-12]. During the same period, in 1994, John Adler at Stanford introduced the CyberKnife system, consisting of a 6-MeV LINAC mounted on a robotic arm with image guidance, which was implemented for clinical use in patients [13,14] (Figure 1).

Radio spectrum and the effects on the human body

The electromagnetic spectrum encompasses a range of frequencies of electromagnetic waves, including the electromagnetic spectrum includes a broad range of electromagnetic wave frequencies, of which high-energy X-rays, gamma rays, and certain subatomic particles are clinically relevant in radiation medicine [15].

Gamma rays and X-rays both consist of high-energy photons exceeding 100 keV, but differ in their origin. In Gamma Knife systems, photon energy is approximately 1.25 MeV, whereas LINAC systems operate with accelerating potentials of around 6 MV. It is important to distinguish MeV (photon energy) from MV (accelerating potential), as these terms are not interchangeable [16].

**Figure 2:**
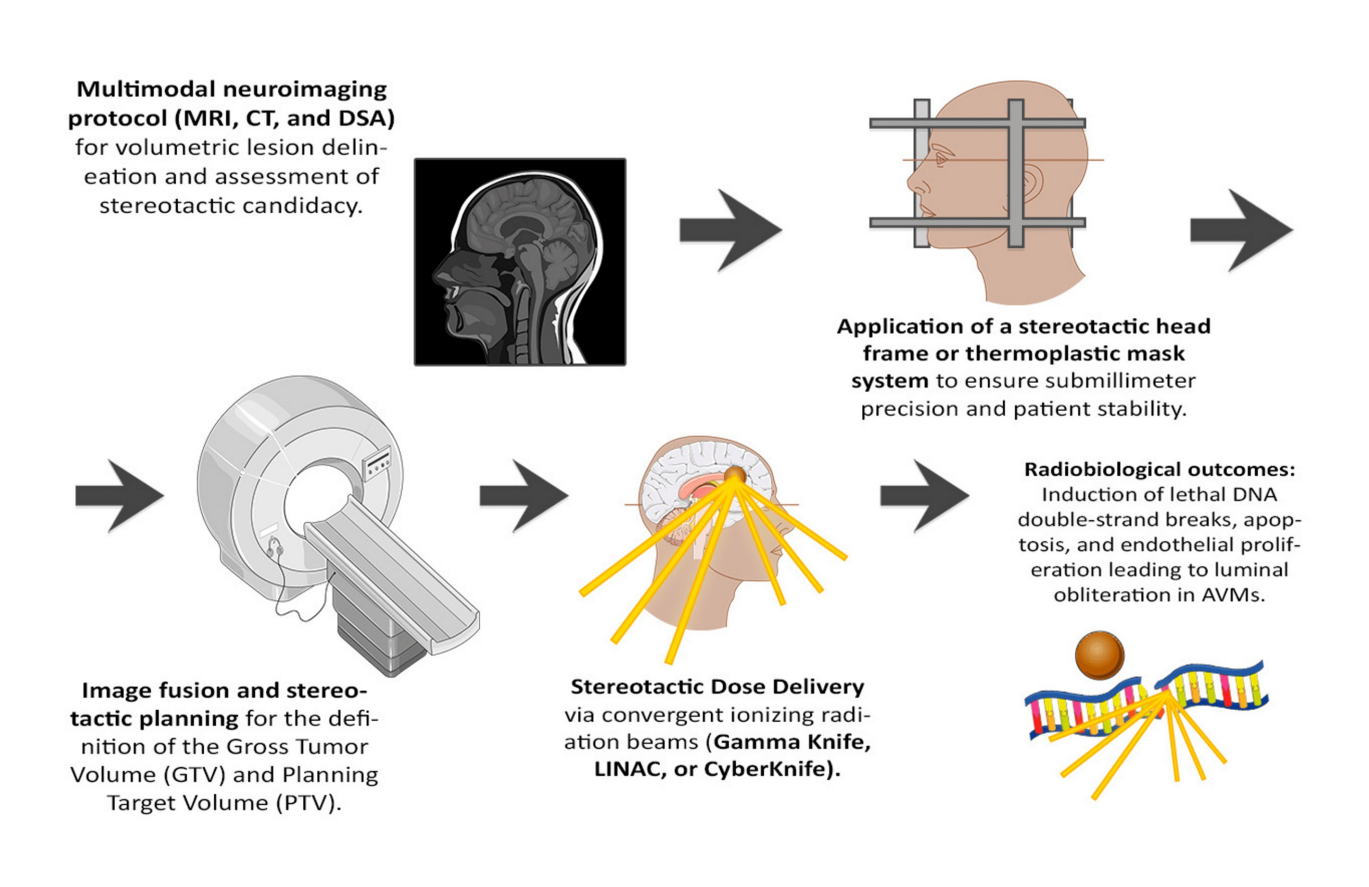
Stereotactic radiosurgery (SRS) process from imaging to biological effect. Conceptual diagram created by the author using Adobe Photoshop 2023 (Adobe Inc., San Jose, CA, USA). Medical illustrations were adapted and modified from Servier Medical Art (https://smart.servier.com/), licensed under CC BY 4.0.

Radiation effects on human tissue are classified as stochastic, which lack a threshold, and deterministic, which occur above a certain dose and may result in cellular damage. In radiosurgery, factors such as penetration depth and the electronic buildup phenomenon are essential for accurate treatment planning [17] (Figure 2).

Technological development and prototypes

These procedures can be performed using different technologies, even though each of them has its advantages and disadvantages. There are four different groups: Gamma Knife, LINAC, CyberKnife, and particle beam radiosurgery. First, fixed-source devices, also known as the Gamma Knife, represent systems designed to ensure maximum precision and safety in radiosurgical procedures. These devices were initially designed by Lars Leksell in Sweden in 1967. The Gamma Knife uses multiple photons of radioactive isotopes, especially cobalt-60. These photons originate from a single source that later converges in an intracranial point to deliver a high focal dose of radiation. The process starts with an adequate selection of the patient with different types of imaging. This can lead to identifying the ideal candidate for the procedure or treatment. After the stereotactic frame is placed, high-resolution magnetic resonance imaging (MRI) sequences are obtained, including T1-weighted, contrast-enhanced T1-weighted, and T2-weighted images. These images are then transferred to the Gamma Knife system, where they are integrated with the stereotactic frame coordinates to accurately localize the target lesion. This process accurately obtained the location of the lesion in the three-dimensional space [18,19].

As previously mentioned, the first Gamma Knife prototype, developed by Leksell and Larsson in 1967 [19], used 179 cobalt-60 sources [20], organized in a hemispheric configuration and aligned through a collimation system that enabled high-precision beam convergence. Through the stereotactic frame, a three-dimensional coordinate system was established, allowing precise localization of the target lesion [21]. This approach facilitated the treatment of pituitary tumors, vestibular schwannomas (VS), arteriovenous malformations (AVMs), and functional disorders. Cobalt-60 decays by emitting beta particles and photons with energies of 1.17 and 1.33 MeV, which are fundamental to its therapeutic application [21]. The second prototype did not introduce major changes, maintaining the use of 179 cobalt-60 sources, although it incorporated a spherical dose distribution profile.

The third prototype, known as “Model B”, was introduced in 1987 and incorporated significant modifications to overcome the limitations of earlier models. It increased the number of cobalt-60 sources and refined the collimation system through improved spherical configuration and interchangeable tungsten collimators, allowing the creation of fields with varying diameters at the focal point. These advancements enhanced positioning accuracy and reduced radiation exposure to surrounding tissues compared with the original device [21].

In 1999, the Gamma Knife “Model C” was introduced, featuring the incorporation of an automatic positioning system (APS). This innovation enabled stereotactic frame movement along the X/Y/Z, improving dose conformity and treatment precision while expanding the mechanical range relative to previous models [22].

In 2006, the Leksell Gamma Knife Perfexion model marked a significant technological advancement. This incorporates, in a differential manner, 192 beams of cobalt-60, compared to past models, arranged in a cylindrical configuration of five concentric rings of collimators of tungsten. This device generated a subdivision with eight sectors, each one with 72 collimators, 24 for each one with the three available diameters: 4,8 and 16 mm. The implementation of this innovative ring system represented a revolutionary change. It allows elimination of the collimator's shells, optimizing precision, security, and the efficiency of the procedure [23].

In 2015, the Leksell Gamma Knife ICON, designed by Elekta, was introduced. The most significant innovation that would bring this model is the implementation of the CBCT system, in conjunction with the table-mounted infrared camera for high-definition motion management during fractionation (HDMM). This permitted the procedure to be performed without the stereotactic frame, using instead thermoplastic mask fixation, which, using the HDMM system, allows monitoring of movement by means of a reflective marker placed on the tip of the patient's nose, providing the option of automatically interrupting radiation if the movement exceeds the threshold [23]. Finally, in the year 2022, the last model of the Leksell Gamma Knife, named Leksell Gamma Knife Elekta Esprit (Elekta AB, Stockholm, Sweden), was introduced.

**Figure 3:**
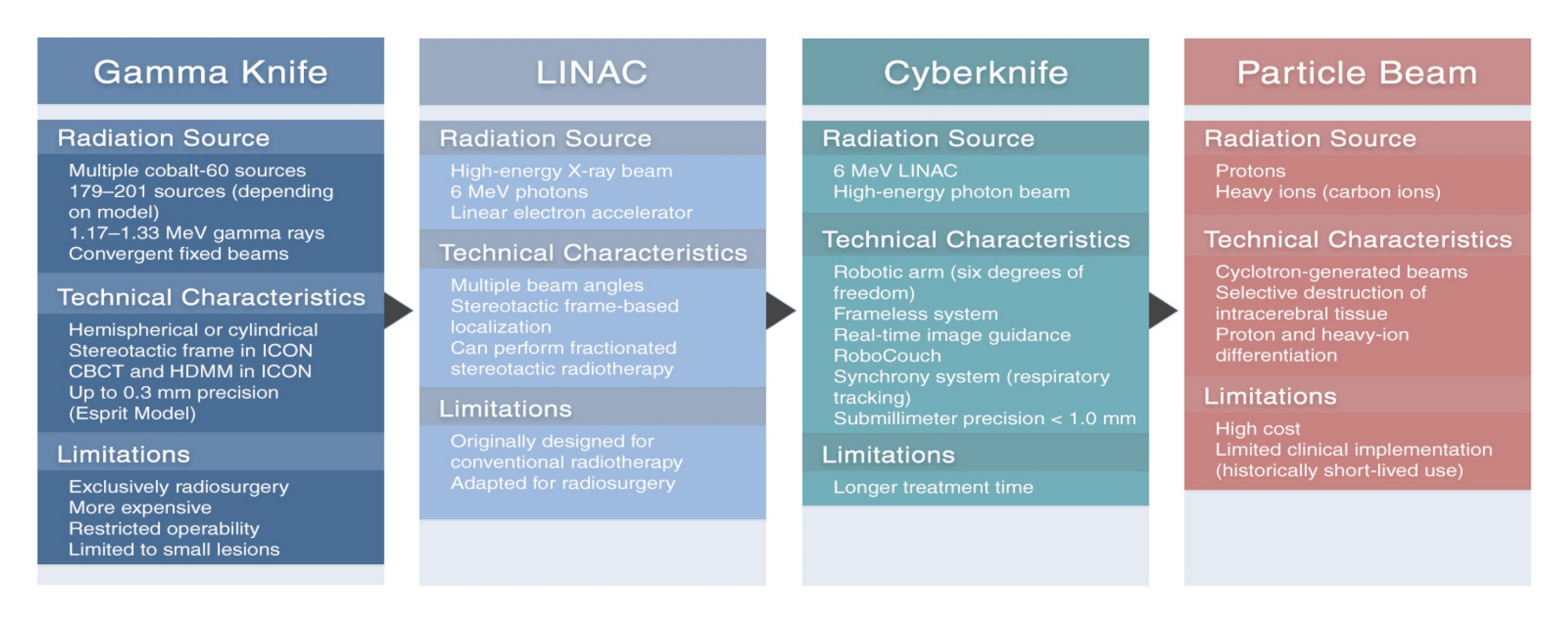
Radiosurgery technologies; Gamma Knife, LINAC, CyberKnife and particle beam compared. Figure created by the authors using Adobe Photoshop 2023 (Adobe Inc., San Jose, CA, USA). LINAC, linear accelerator; CBCT: cone-beam computed tomography; HDMM, high-definition motion management during fractionation (HDMM).

The CyberKnife is a frameless, stereotactic robotic radiosurgery system that combines a LINAC with a six-degree-of-freedom robotic arm, coupled to a real-time imaging system. This allows the high-energy photon beam to be directed from multiple non-coplanar angles. The CyberKnife can be used in brain lesions, but also in other malignant lesions throughout the body. This system integrates a robotic couch (RoboCouch, Accuray Inc., Madison, WI, USA), which allows for precise patient alignment during the procedure, and various interchangeable collimators, making it possible to adapt the beam shape to the volume of the tumor lesion [23] (Figure 3).

Clinical indications

Radiosurgery is also used as an adjuvant to the main treatment when the lesions cannot be completely resected. When there is a larger lesion, an initial session of radiotherapy can be performed to reduce its size, or when complete resection is not possible, and the remaining lesion needs to be irradiated. Therefore, the following are the most frequent indications for the use of radiosurgery.

Brain Metastases

Brain metastases (BM) are the most common intracranial tumors and occur in approximately 20% of patients with extracranial malignancies [24]. These lesions can produce significant neurological deficits depending on their size, location, and associated mass effect.

Hypofractionated SRS delivers radiation in multiple sessions, allowing improved tumor control while reducing toxicity to surrounding brain tissue. Current clinical guidelines support its use in selected patients with BM, offering favorable local control and neurological preservation compared to conventional approaches [24].

Historically, treatment relied on whole-brain radiotherapy (WBRT); however, current clinical evidence demonstrates that SRS offers comparable survival rates while preserving the patient's neurocognitive function. For this reason, guidelines from the American Society for Radiation Oncology (ASTRO) prioritize the use of SRS to minimize treatment-related toxicity [25].

Vestibular Schwannomas

Vestibular schwannomas (VS) are benign tumors of the vestibulocochlear nerve and represent the most common lesions of the cerebellopontine angle. SRS is recommended for tumors ≤3 cm, particularly those <2.5 cm, with the objective of achieving long-term growth control while preserving neurological function. Tumor control rates approach 90% at 10 years, with lower morbidity compared to conventional surgery [26].

Arteriovenous Malformations

Arteriovenous malformations (AVMs) are non-neoplastic vascular lesions characterized by direct arterial-to-venous shunting without an intervening capillary bed. Clinically, they may present with hemorrhage or seizures, which largely determines the management strategy. Surgical resection is often preferred in cases of hemorrhagic presentation. However, stereotactic radiosurgery (SRS) is indicated for AVMs located in surgically inaccessible regions or measuring approximately 2.5-3 cm. Obliteration rates range from 70% to 80% in appropriately selected lesions but decrease in larger malformations [27].

Meningiomas

Meningiomas are the most common primary intracranial tumors. Although typically benign and slow-growing, treatment is indicated in symptomatic, atypical, or progressively enlarging lesions. Surgical resection remains the first-line therapy; however, stereotactic radiosurgery (SRS) is preferred for medium-sized tumors in surgically high-risk locations. Long-term tumor control rates with SRS exceed 90%. SRS is also used as adjuvant therapy after subtotal resection or as salvage treatment for recurrence, although control rates may be lower compared with primary radiosurgical management [28].

Refractory Epilepsy

Approximately 30% of patients with epilepsy develop drug-resistant seizures, requiring alternative therapeutic strategies. Surgical resection of the epileptogenic zone, particularly anterior temporal lobectomy, remains the preferred approach in appropriate candidates.

Stereotactic radiosurgery (SRS) has emerged as a non-invasive alternative, especially in medial temporal lobe epilepsy. Although seizure control rates are generally lower than those achieved with resective surgery, SRS may be considered in patients with high surgical risk, lesions located in eloquent areas, recurrence after prior surgery, or refusal of invasive procedures [29].

Pituitary Adenomas

Pituitary adenomas (PAs) represent 10-15% of primary intracranial tumors and are classified by size (microadenomas ≤1 cm and macroadenomas ≥1 cm) and hormonal activity (functional and non-functional) [30]. Transsphenoidal resection remains the first-line treatment for symptomatic lesions. Gamma Knife radiosurgery (GKRS) is primarily indicated for residual or recurrent adenomas, providing focused radiation with millimetric precision while preserving adjacent structures. Its objectives include tumor growth control, hormonal normalization, and preservation of pituitary function [30].

Refractory Trigeminal Neuralgia

**Figure 4:**
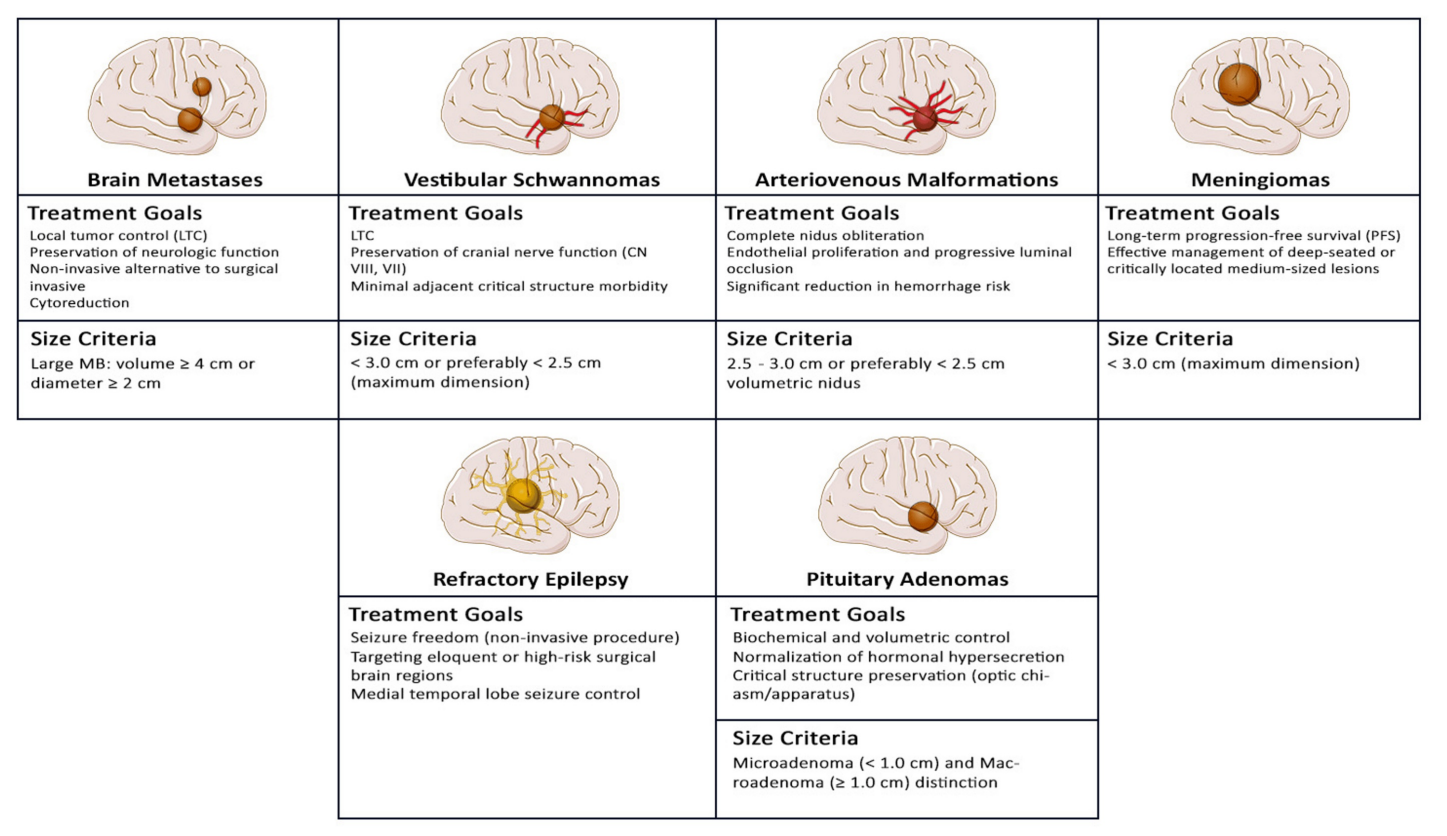
SRS clinical indications by pathology. Conceptual diagram created by the author using Adobe Photoshop 2023 (Adobe Inc., San Jose, CA, USA). Medical illustrations were adapted and modified from Servier Medical Art (https://smart.servier.com/), licensed under CC BY 4.0.

Trigeminal neuralgia is the most common craniofacial pain syndrome and presents as sudden episodes of severe facial pain within the distribution of one or more trigeminal nerve branches, frequently associated with neurovascular compression at the root entry zone. Initial treatment is pharmacological; however, refractory cases require surgical intervention [31]. When medication fails, options include microvascular decompression, percutaneous procedures, and radiosurgery. While decompression offers durable pain relief but carries surgical risks, radiosurgery creates a precisely targeted lesion with good outcomes and a low complication rate, although recurrence may occur [31] (Figure 4).

## Conclusions

Since its inception as a specialized therapeutic modality, SRS has become one of the fundamental pillars of modern neurosurgery and oncology. It has evolved into a standard treatment option supported by international clinical guidelines, providing patients with effective disease control and a significantly lower risk profile compared to conventional surgery. We are currently in an era of technological progress driven by the integration of artificial intelligence (AI) and high-definition neuroimaging. This synergy acts as the engine for personalized medicine, where each treatment plan is precisely tailored to the biological and radiological profile of every patient. Such constant evolution ensures that radiosurgery will continue to exceed the limits of safety and efficiency, offering increasingly individualized care for the future.
